# Beta2-Integrins and Interacting Proteins in Leukocyte Trafficking, Immune Suppression, and Immunodeficiency Disease

**DOI:** 10.3389/fimmu.2019.00254

**Published:** 2019-02-19

**Authors:** Susanna C. Fagerholm, Carla Guenther, Marc Llort Asens, Terhi Savinko, Liisa M. Uotila

**Affiliations:** ^1^Molecular and Integrative Biosciences Research Program, Faculty of Bio- and Environmental Sciences, University of Helsinki, Helsinki, Finland; ^2^Iho- ja Allergiasairaala, HUS, Helsinki, Finland; ^3^Research Services, University of Helsinki, Helsinki, Finland

**Keywords:** integrin, trafficking, kindlin-3, leukocyte adhesion deficiency, leukocyte adhesion cascade

## Abstract

Beta2-integrins are complex leukocyte-specific adhesion molecules that are essential for leukocyte (e.g., neutrophil, lymphocyte) trafficking, as well as for other immunological processes such as neutrophil phagocytosis and ROS production, and T cell activation. Intriguingly, however, they have also been found to negatively regulate cytokine responses, maturation, and migratory responses in myeloid cells such as macrophages and dendritic cells, revealing new, and unexpected roles of these molecules in immunity. Because of their essential role in leukocyte function, a lack of expression or function of beta2-integrins causes rare immunodeficiency syndromes, Leukocyte adhesion deficiency type I, and type III (LAD-I and LAD-III). LAD-I is caused by reduced or lost expression of beta2-integrins, whilst in LAD-III, beta2-integrins are expressed but dysfunctional because a major integrin cytoplasmic regulator, kindlin-3, is mutated. Interestingly, some LAD-related phenotypes such as periodontitis have recently been shown to be due to an uncontrolled inflammatory response rather than to an uncontrolled infection, as was previously thought. This review will focus on the recent advances concerning the regulation and functions of beta2-integrins in leukocyte trafficking, immune suppression, and immune deficiency disease.

## Integrins and integrin regulation

Integrins are heterodimeric type I transmembrane proteins consisting of alpha and beta subunits (1). Integrins are expressed in all nucleated cells and play a key role in adhesion, cell communication, and migration. They mediate adhesion to the extracellular matrix, by binding to the RGD motif of fibronectin, collagen, and laminin, among others (2). Integrins in leukocytes also bind to soluble ligands such as the complement component iC3b, and to other cells, by binding to ICAMs (Intercellular adhesion molecules) and VCAM-1 (Vascular cell adhesion molecule) (3, 4). Additionally, integrins link to the actin cytoskeleton inside the cell and thereby connect the inside of the cell with the outside.

Integrins have large extracellular domains which contain the ligand-binding sites, and short cytoplasmic domains which are important for integrin regulation. The ability of the integrin to bind to ligands is regulated through conformational changes as well as by integrin clustering. Integrins can be found in three main conformational states: inactive (bent-closed), intermediate (extended-closed), and active state (extended-open) (5). The predominant state seems to be the inactive (bent-closed) state based on affinity and thermodynamics studies with K562 cells (alpha5beta1-integrins, bent-closed: 99.75%; extended-closed: 0.10%; extended-open: 0.15%) (6). The active conformation (extended-open) has a 4,000-fold increase in ligand affinity compared to the other two states (7). Also on resting peripheral T cells the vast majority of LFA-1 (Lymphocyte function-associated antigen-1, alphaLbeta2-integrin) appear to be in the inactive conformation, as stabilizing the active conformation leads to a 1,000-fold increase in affinity of the integrin (8). The LFA-1 conformational change (integrin extension) on the surface of migrating T cells has recently been directly measured by super-resolution microscopy [interferometric photoactivation, and localization microscopy (iPALM)] (9).

Integrin activation takes place upon cell stimulation through various cell surface receptors such as chemokine receptors or the T cell receptor. Cell stimulation triggers an inside-out signaling pathway that ultimately recruits cytoplasmic factors such as talin and kindlin to the NPxY motifs of the cytoplasmic tail of the integrin's beta-chain, which causes the cytoplasmic tails of the integrin subunits to separate (10) and switches the integrin to the active (extended-open) conformation (11, 12). Kindlin and talin connect the integrin to the actin cytoskeleton and stabilize the extended-open conformation of the integrin through actin cytoskeleton exerted tensile force (6, 13). In addition, many other proteins, such as 14-3-3 proteins, alpha-actinin, coronin 1A, cytohesin 1, filamin A, and Dok1 can interact directly with the integrin beta-chain and modulate integrin function (14–16). These interactions are often regulated by phosphorylation of the integrin beta-chain cytoplasmic domain (15–18).

In addition to their ability to respond to the environment through inside-out signaling, integrins can take part in a variety of signaling cascades following ligand binding (outside-in signaling). Integrins take part in the formation of adhesion complexes and focal adhesions in cells such as fibroblasts, modulation of actin cytoskeleton dynamics, cell migration, differentiation, proliferation, angiogenesis, and apoptosis (19).

## Beta2-Integrins in Leukocyte Trafficking

Beta2-integrins (CD11a/CD18, alphaLbeta2, LFA-1; CD11b/CD18, alphaMbeta2, Mac-1, CR3; CD11c/CD18, alphaXbeta2, p150.95, CR4; and CD11d/CD18, alphaDbeta2) are a subgroup of integrins which share a common beta2- or CD18-chain but have different alpha-chains and ligands. Beta2-integrins are expressed exclusively in leukocytes, but the different members have their own distinct expression pattern. CD11a/CD18 is expressed on all leukocytes, while CD11b/CD18, CD11c/CD18, and CD11d/CD18 are mainly expressed on myeloid cells, but at varying levels (19, 20). CD11a/CD18 has a more restricted ligand binding capacity than the other beta2-integrins, and binds ligands such as ICAM-1-5 found on the surface of other cells. In contrast, CD11b/CD18 is a very promiscuous integrin with more than 40 reported ligands, including ICAMs, iC3b, fibrinogen, RAGE (receptor for advanced glycation end products), and CD40L (20). Interestingly, ligand-specific blockade of CD11b/CD18 has recently been shown to protect against bacterial sepsis, while blocking all CD11b/CD18 functions potentiates it, showing that CD11b/CD18 indeed has very complicated roles in immunity due to its many ligands (21). In addition to leukocytes, beta2-integrins are also found in extracellular vesicles (EVs), and integrins in EVs may play novel roles in development of pathogenic conditions such as sepsis (22).

**Figure 1:**
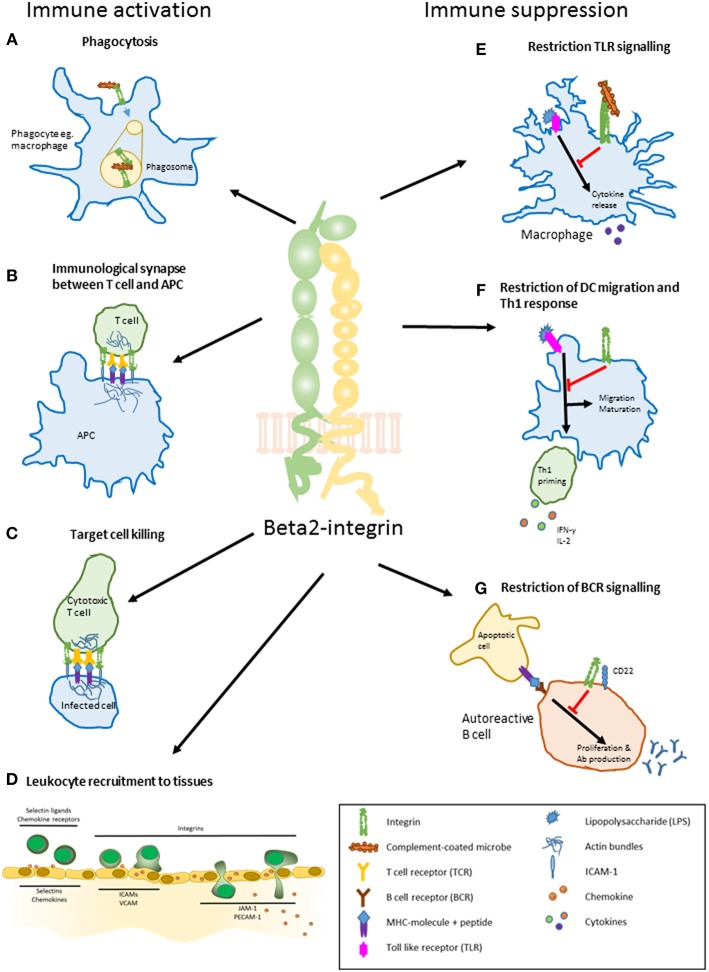
The main roles of beta2-integrins in immune activation and suppression. **(A)** phagocytosis. Beta2-integrins mediate phagocytosis by binding to iC3b on the surface of complement-coated bacteria. **(B)** regulation of T cell activation by a dendritic cell. Beta2-integrins participate in the formation of an immunological synapse between a T cell and an antigen presenting cell such as DC. The synapse stabilizes the interaction between and regulates signaling in the two participating cells. **(C)** target cell killing. Beta2-integrins participate in forming and maintaining an immunological synapse between a cytotoxic T cell and an infected cell. **(D)** leukocyte recruitment to tissues. Leukocytes are activated by selectins and chemokines on the surface of activated endothelial cells close to a site of inflammation. This slows down the leukocyte speed and induces integrins to change their conformation through inside-out signaling, allowing them to bind ICAMs on the endothelium. Beta2-integrins are essential in the slow rolling and firm adhesion of a leukocyte, after which the cells transmigrate to the inflamed tissue. Leukocytes use the chemokine gradient to navigate toward the site of inflammation. **(E)** regulation of TLR-signaling. Beta2-integrin CD11b/CD18 restrains macrophage activation and cytokine production upon TLR (Toll-like receptor) activation by LPS (lipopolysaccharide). **(F)** restriction of dendritic cell migration, maturation, and Th1 priming. Proper beta2-integrin—cytoskeleton linkage controls DC maturation toward a migratory phenotype and restricts priming of Th1 cells. **(G)** restriction of B cell receptor signaling. Interaction of CD11b/CD18 and CD22 on the surface of an autoreactive B cell leads to constraint in BCR signaling. This decreases auto-reactive B cell proliferation and antibody production.

It is undisputed that beta2-integrins are of fundamental importance for leukocyte trafficking. This is because they are required for the firm adhesion to the endothelial layer surrounding the blood vessels under conditions of shear flow (blood flow) and for leukocyte extravasation into tissues (23). The leukocyte adhesion cascade (Figure 1) is a multistep process involving rolling, firm adhesion or arrest, spreading/crawling, and finally extravasation (24). This complex process is accomplished by several proteins acting in parallel and succession, as the leukocyte proceeds to its destination. Initially contacts between the leukocyte and the endothelial cells allows selectins and ICAM-1 on endothelial cells to mediate leukocyte rolling on the endothelium. The close contact between the cells during rolling allows the leukocyte to sense chemokines present on the endothelium. In neutrophils, both selectins and chemokine receptors activate beta2-integrins via a signaling pathway involving the small GTPase Rap1a and phosphatidylinositol-4-phosphate 5-kinase (PIP5Kγ90). The activation of beta2-integrins involves conversion into the intermediate affinity state that mediates slow rolling, followed by conversion into the high affinity state, which mediates leukocyte arrest (25). Both selectins and integrins can form slip bonds, whose lifetime is shortened by applied shear force, as well as catch bonds, which strengthen under shear force (26–28), inducing further changes downstream of the integrins. During these leukocyte-endothelial contacts numerous integrin-ligand bonds are continuously broken and formed and further reinforced by the recruitment of more integrins and downstream cytoskeletal proteins such as talin, kindlin-3, focal adhesion kinase, and paxillin to form adhesion complexes which strengthen cell adhesion and induce actin reorganization and cell spreading (26). Following adhesion, cells crawl along the endothelium looking for a suitable extravasation site, a process critically dependent on the beta2-integrin CD11b/CD18 (29). As integrins act as mechanosensors in cells (30), it is likely that integrins are also central for the subsequent steps of probing the endothelium for suitable points of exit, either through a paracellular or transcellular route.

Talin has long been known to be indispensable for leukocyte trafficking (31–34). More recently, also kindlin-3 and its interaction with the beta2-integrin tail has been shown to be vital for neutrophil and effector T cell firm adhesion under shear flow and for neutrophil and T cell trafficking *in vivo* (35–38), and for homing of progenitor T cells to the vascularized thymus (39). However, talin and kindlin-3 regulate different aspects of leukocyte trafficking. Talin is required for the conformational change of the integrin to the extended, intermediate affinity conformation which mediates slow rolling. In contrast, both talin and kindlin-3 are required for the induction of the high-affinity conformation, full integrin activation and neutrophil arrest (33, 38, 40). Recently, Src kinase-associated phosphoprotein 2 (Skap2) has been shown to be essential for the recruitment of talin and kindlin-3 to the beta2-integrin tail, and for neutrophil trafficking *in vivo* (41). Interestingly, a bent-open conformation of beta2-integrins has been reported on neutrophils, which limits neutrophil recruitment by binding to ICAM-1 *in cis*, but the molecular mechanisms regulating this process are currently unknown (42).

In contrast to talin and kindlin-3, filamin A has been suggested to negatively regulate integrin functions *in vitro* (15, 43, 44). However, it has also been reported to be required for platelet shear flow adhesion because it stabilizes the links between the plasma membrane and the underlying actin cytoskeleton (45). Recent studies utilizing T cell-specific filamin A-deficient mice have shown that filamin A is required for the optimal firm adhesion of T cells under shear flow conditions, trafficking of T cells into lymph nodes, and to the inflamed skin (46). These results demonstrate that in T cells, filamin A does not function as an integrin inhibitor but rather is required for cell trafficking *in vivo*. However, filamin A is not required for neutrophil adhesion under shear flow conditions, but instead filamin A-deficient neutrophils display enhanced adhesion, spreading, and defects in uropod retraction, thereby revealing cell-type specific functions of this integrin interacting protein (47, 48).

In contrast to leukocyte trafficking from blood stream to lymph nodes and tissues, leukocyte trafficking within tissues (e.g., in a confined 3D environment in the absence of shear flow) is a mechanistically different process that can occur even in the absence of integrins (49). In lymph nodes, integrins, and chemokine receptors contribute partly to naïve T cell migration speed (50). In this environment the integrin CD11a/CD18 (LFA-1) is required as a frictional interface with the substrate (the so called “integrin clutch”) by generating traction forces, but does not mediate substantial adhesion to the substrate (50). In some cases, integrins can even *restrict* leukocyte migration in tissues. Indeed, the beta2-integrin-kindlin-3 interaction negatively regulates DC migration to lymph nodes both under steady state and inflammatory conditions (36, 51). beta2-integrins restrict DC migration through a downstream mechanism which involves regulation of the transcriptional program and migratory phenotype of these cells (Figure 1).

## Beta2-Integrins in Other Immune-Related Functions

In addition to their fundamentally important role in leukocyte trafficking, beta2-integrins also mediate other cell-cell contacts that are essential for immunological processes (Figure 1). Beta2-integrins (e.g., CD11a/CD18-integrin; LFA-1) are central components of the immunological synapse which forms between an antigen presenting cell (APC) and a T cell [reviewed in Dustin (52)], between a B cell and a T cell (53) and between an NK cell and its target cell (54). In brief, the cell-cell interactions mediated by CD11a/CD18 on the T cell enables T cell activation, by binding to ICAM-1 on the APC. T cells sample antigens on dendritic cells in lymph nodes via short term contacts, termed kinapses (52). When antigen is found, T cells stop migrating and form an immunological synapse with the dendritic cell (52). LFA-1 on the T cell binding to ICAM-1 on the DC play a crucial role in this structure. LFA-1, together with talin, kindlin-3, and Rap1, is positioned in the p-SMAC (peripheral supramolecular activation cluster), thereby stabilizing the interaction between the T cell receptor and peptide-MHC II at the center of the contact (c-SMAC) (52, 55). Optimal T cell activation *in vivo* requires talin and kindlin-3 to bind to LFA-1 (32, 56). Upon activation, LFA-1 can the signal into the T cell and thereby contribute to T cell activation and polarization of the T cell response (57). For example, LFA-1 ligation in T cells has been shown to promote Th1 polarization through a pathway involving Erk and Akt-mediated GSK3beta-inhibition, in turn leading to activation of the Notch pathway (58), and LFA-1 can also be regulated by, and engage in crosstalk with TGF-beta signaling in T cells (59, 60). In addition, a role for an intracellular pool of beta2-integrins in T cell activation and differentiation has recently been reported (61).

In addition to T cell activation, CD11a/CD18 is involved in the killing of infected target cells by cytotoxic T cells, by stabilizing the contact between the T cell and the target cell, and by sealing the contact zone so that cytolytic granules cannot escape (57). LFA-1 furthermore plays a role in the generation of T cell memory (57), survival of T follicular helper cells (62) and regulatory T cells (63) and B cell-mediated antibody production, by mediating cell-cell contacts, but also by initiating intracellular signaling cascades (57, 64). LFA-1 is important for CD8+ T cell trafficking (65) and for Th2 (but not Th1) homing, as well as Th2-induced allergic lung disease (66). Interestingly, certain CD11a polymorphisms critically influence Th2 homing (67).

In myeloid cells such as macrophages, beta2-integrins can initiate intracellular signaling pathways leading to cytokine secretion, either by themselves or together with Toll like receptors (TLRs) (21, 68, 69). In addition, many neutrophil functions such as cytokine release and oxidative burst are dependent on beta2-integrins (70–73). CD11b/CD18 and CD11c/CD18 are receptors for complement component iC3b and are essential for phagocytosis of opsonized pathogens in neutrophils and other phagocytic cells, where they induce a RhoA-dependent phagocytic pathway (74–76). The differential roles of these two highly similar integrins have been studied *in vivo* in CD11b^−/−^ and CD11c^−/−^ mice. The results indicate that CD11b/CD18 is involved in neutrophil functions and in the anti-inflammatory functions of macrophages, whereas CD11c/CD18 is more relevant in the regulation of macrophage inflammatory functions (77). Recently, beta2-integrins has been shown to be required for recruitment of monocytes, as well as hematopoiesis of these cells during Schistosome infection, and a low expression of beta2-integrins correlates with increased parasite burden in a murine model of the disease (78).

## Beta2-Integrins in Immune Suppression

In addition to their well-characterized role in mediating cellular interactions and promoting pro-inflammatory signaling, beta2-integrins have also been associated with many immunosuppressive functions (20) (Figure 1). Beta2-integrins can inhibit TLR signaling in macrophages through negative feedback loops, either directly or indirectly, through the anti-inflammatory cytokine IL-10 (79, 80). TLR stimulation leads to PI(3)K- and RapL-mediated inside-out activation of CD11b/CD18. Integrin outside-in signaling activates Src/Syk, leading ultimately to degradation of the important TLR signaling transducers MyD88 and TRIF and downregulation of TLR signaling (80). The mechanism of CD11b/CD18-dependent modulation of TLR responses has been shown to involve inhibition of the NF-κB pathway and activation of the p38 MAPK pathway (81). Beta2-integrins have been found to repress DC-mediated T cell activation (82–84), and the presence of CD11b/CD18 on APCs has been demonstrated to suppress Th17 differentiation and lead to immune tolerance (85, 86). Recently, CD11b/CD18-expressing neutrophils have been shown to suppress T cell-dependent influenza pathology *in vivo* by limiting T cell proliferation (87). The immunoregulatory role of leukocyte integrins may be taken advantage of by the macrophage-infecting bacterium *Francisella tularensis*, which is phagocytosed in a CD11b-dependent manner and uses the CD11b-driven inhibition of inflammasome activation to evade the innate immune system (88). In addition to opsonized bacteria, CD11b/CD18, and CD11c/CD18 also recognize iC3b-opsonized apoptotic cells, which leads to inhibition of proinflammatory cytokine production through NF-κB inhibition (89).

A series of important findings of the immunoregulatory roles of beta2-integrins has been produced using mice where the kindlin-3 binding site in the CD18-chain has been mutated, leading to expressed but inactive integrins on the surface of immune cells (TTT/AAA-beta2-integrin KI mice) (35). DCs from these mice mature toward a migratory phenotype, accumulate in lymphoid organs, and induce increased Th1 immune responses *in vivo* (51). In addition, functional integrins are essential for restricting the accumulation of mast cells in inflamed skin and mast cell responses *in vitro*, and inflammatory cytokine production in the inflamed skin *in vivo* (36). In the context of obesity-associated inflammation, mice on a high fat diet display increased numbers of neutrophils in white adipose tissue, increased insulin resistance and elevated inflammatory profile (90). However, the total deletion of an individual beta2-integrin, e.g., CD11b in mice led to increased weight gain on a high fat diet and lowered insulin sensitivity but to decreased inflammatory gene expression compared to WT mice *in vivo*, suggesting that the CD11b-integrin specifically is pro-inflammatory under diet-induced obesity conditions (91).

Interestingly, variations at the ITGAM gene, which encodes for CD11b, is one of the strongest genetic risk factors for systemic lupus erythematosus (SLE). These nucleotide polymorphisms confer amino acid changes in the CD11b protein, leading to deficient ligand binding, and a reduced ability to restrict cellular cytokine expression (92–94). Interestingly, activation of CD11b/CD18 with a CD11b agonist LA1 is able to overcome the effects of CD11b/CD18 malfunction in the carriers of the SLE-associated polymorphisms (95).

While most of the findings concerning the immunoregulatory role of beta2 integrins have been made in myeloid cells, also some lymphocyte subgroups express CD11b/CD18. Indeed, in B cells, CD11b/CD18 has been shown to negatively regulate B cell receptor signaling to maintain autoreactive B cell tolerance (96). Together, these results show that, while it is clear that beta2-integrins are important for immune cell activation and function, beta2 integrins (especially CD11b) have an equally significant role in repressing the body's reactions against self. Therefore, manipulating integrin activation pharmacologically could be an efficient therapeutic approach in treating certain inflammatory or autoimmune diseases.

## Leukocyte Adhesion Deficiencies

The importance of beta2-integrins in immunity is highlighted by the rare genetic diseases known as Leukocyte adhesion deficiencies type I and type III (LAD-I and LAD-III) (Table 1). LAD syndromes are a group of congenital autosomal-recessive diseases with immune deficiency condition resulting in impaired leukocyte adhesion and migration. In LAD-I, the expression of CD18 (the beta2-integrin-chain) is either diminished or abolished. In LAD-III, mutations in kindlin-3 prevents it from activating beta2-integrins. Both conditions present partly with similar symptoms, which include leukocytosis and a lack of neutrophil extravasation from the blood stream into tissues. Consequently, the patients end up suffering from recurrent life-threatening infections, unless they receive a hematopoietic stem cell transplant (HSCT) (97). LAD-II is a selectin- (rather than integrin) related disease which is caused by a failure in selectin ligand expression (98) and will not be discussed further here.

**Table 1:** Beta2-integrins in immunodeficiency and inflammatory disease.

**Disease**	**Symptoms**	**Beta2-integrin defects**	**Impaired immune functions**
LAD I	Bacterial and fungal infections in skin and other tissues; Delayed wound healing. Periodontitis, Leukocytosis, Candidiasis	Mutation in CD18 chain leading to decreased or non-existent expression of beta2-integrins	Decreased neutrophil trafficking to the site of inflammation. Defective adaptive immune responses (especially in T cells) Impaired restriction of inflammatory responses (e.g., cytokine release)
LAD III	Same as Lad I but also Glanzmann thrombasthenia. Osteopetrosis	Mutation in kindlin-3 protein, leading to incorrect activation of betal-, beta2-, and beta3-integrins	In addition to LAD I functions: Impaired platelet activation and blood clotting Impaired osteoclast function
SLE (Systemic Lupus Erythematosus)	Severe fatigue, Joint pain and swelling, Headaches, Rashes on cheeks and nose, Hair loss, Anemia, Blood-clotting problems	R77H, P1146S and A858V substitutions in CDllb	Impaired ligand binding and phagocytosis(R77H) Increased adhesion, spreading, and migration (P1146S) Increased pro-inflammatory cytokine release (R77H and P1146S)

**LAD I**—Over 200 mutations have been identified in LAD-I patients which cause decreased expression of CD18. The severity of the disease varies according to the functionality of the beta2-integrin (99). LAD-I patients suffer from life threatening bacterial and fungal infections early in life, and especially neutrophil trafficking is reduced into the inflamed tissue. In a recent (2018) review of all published LAD cases before 2017 (323 cases) (100) it was reported that the most common infections in severe LAD-I (<2% CD18 expression) were respiratory tract infections (including pneumonia), sepsis, and otitis media whilst in LAD-I with moderate CD18 expression the most common infections were periodontal infection, otitis media and sepsis. Perianal skin infections and necrotic skin ulcers were reported in both groups. Delayed umbilical cord detachment is common. In addition, patients suffer from symptoms such as delayed wound healing.

For severe LAD-I, survival beyond 2 years of age was only 39%, showing that severe LAD-I remains a life-threatening condition (100). The prognosis for LAD-I with moderate CD18 expression is much better, with survival over 2 years and beyond (to adulthood) for over 90% of cases with >4% CD18 expression (100, 101).

HSCT remains the only cure for patients expressing very low (<1–2 %) levels of CD18 protein in leukocytes, but unfortunately transplant-related mortality remains high (19% for all groups in LAD-I) (100).

Beta2-integrin deficient mice have similar immune defects as LAD-I patients (102). These mice have been useful to investigate the role of beta2-integrins and their function in different leukocytes (102, 103).

**LAD-III—**LAD-III is a much rarer disease than LAD-I, with <40 patients reported worldwide (104). Patients suffer from similar symptoms as LAD-I patients, e.g., recurrent bacterial infections including bacteremias, pulmonary infections, omphalitis, and other soft tissue infections. Also fungal infections have been reported. However, unlike LAD-I patients, LAD-III patients additionally have Glanzmann type thrombasthenia, a bleeding disorder. Transfusions have been performed in >90% of cases as bleeding is a hallmark of the disease and remains a serious complication (105). In addition, recombinant factor VIIa has been used successfully in LAD-III to treat bleeding events (105). Furthermore, patients can suffer from osteopetrosis, due to deficient integrin-mediated osteoclast bone resorption.

LAD-III patients have normal integrin expression but carry mutations in the FERMT3 gene encoding kindlin-3 protein (106). Since kindlin-3 binds to beta1, beta2, and beta3-integrins and regulates their function, patients display more complex symptoms compared to LAD-I patients. In platelets kindlin-3 is required for αIIbβ3-integrin-mediated formation of blood clots. Kindlin-3 further regulates normal bone regeneration by several integrins. As for LAD-I, the only curative treatment for LAD-III is HSCT, and HSCT-related mortality remains high [22%, (105)].

Kindlin-3 has a central role in immunity which is shown by the phenotype of the kindlin-3 deficient mice (12, 38). These mice die early after birth because of excessive bleeding. These mice, as well as mice carrying a mutation in the kindlin-3 binding site in beta2-integrin cytoplasmic tail (TTT/AAA-beta2-integrin KI mice) have shown a crucial role of kindlin-3 and beta2-integrins in the regulation of immune cells (35, 36, 51, 56).

## LAD and Inflammation

Many of the symptoms in LAD patients are thought to be caused by defective leukocyte (especially neutrophil) trafficking into inflamed tissue. However, not all symptoms in LAD-I are due to defective leukocyte-mediated immune surveillance. Instead, periodontitis and associated bone loss in LAD-I has recently been shown to be associated with an increased inflammatory response, with excessive production of IL-17 and related cytokines (107), and blocking the IL-17 cytokine response reduces symptoms in a LAD-I patient (108). In addition, particular inflammatory disorders (e.g., colitis) have been reported in LAD-patients (109–111). This indicates that at least some pathological symptoms in LAD-I patients are caused by dysregulated inflammatory responses. The increased IL-17 production in LAD-I patients may be—at least in part—due to defective neutrophil recruitment into tissues e.g., dysregulation of the so called “neutrostat,” which senses and regulates neutrophil numbers *in vivo* (107). However, beta2-integrins have been shown to *directly* restrict cytokine responses in many types of immune cells, such as macrophages (80), DCs (51), and mast cells (36), and to restrict Th1 (51) and Th17 (85) polarization *in vivo*. In addition, functional beta2-integrins restrict expression of cytokines in a skin inflammation model, although neutrophil trafficking is relatively normal in this model (36). Dysregulated cytokine responses may therefore contribute to the paradoxical increase in inflammation (periodontitis, colitis) in LAD-I patients (107, 109, 110, 112).

## Therapeutic Targeting of Beta2-Integrins

Because of the crucial role of beta2-integrins in leukocyte functions such as leukocyte recruitment, beta2-integrins have been considered attractive targets in inflammatory disease such as psoriasis, arthritis, and multiple sclerosis [reviewed in Mitroulis et al. (113)]. Indeed, an antibody against alphaL-integrin chain, efalizumab, has previously been in clinical use in psoriasis (113). However, the drug was withdrawn from the market in 2009 because it was associated with serious side effects, e.g., reactivation of latent John Cunningham (JC) virus infection and resulting progressive multifocal leukoenphalopathy (PML). Therefore, therapeutic blocking of beta2-integrins in disease may be difficult because these molecules play such multifaceted roles in central immune reactions.

## Conclusions and Future Perspectives

Beta2-integrins are of crucial importance for leukocyte trafficking and immune cell activation, but interestingly play a role in immune suppression as well. Consequently, dysfunctional or absent integrins are linked not only to immune deficiency disease but also to inflammatory disease, thereby contributing to both ends of the spectrum of immune-related diseases. A better understanding of the disease processes where dysfunctional beta2-integrins are involved may provide novel drug targets for immunodeficiency and inflammatory disease symptoms (95, 108).

## Author Contributions

All authors listed have made a substantial, direct and intellectual contribution to the work, and approved it for publication.

### Conflict of Interest Statement

The authors declare that the research was conducted in the absence of any commercial or financial relationships that could be construed as a potential conflict of interest.
